# How different are offline and online diplomacy? A comparative analysis of public statements and SNS posts by delegates to the United Nations

**DOI:** 10.3389/fdata.2024.1304806

**Published:** 2024-04-08

**Authors:** Takuto Sakamoto, Momoko Araki, Hiroto Ito, Tomoyuki Matsuoka

**Affiliations:** ^1^Graduate School of Arts and Sciences, University of Tokyo, Tokyo, Japan; ^2^Institute for Digital Observatory, University of Tokyo, Tokyo, Japan

**Keywords:** international relations, digital diplomacy, United Nations, SNS, sentiment analysis, topic modeling, word embedding

## Abstract

**Introduction:**

This article investigates the evolving landscape of diplomacy in the digital age, focusing on diplomats at the United Nations (UN) Headquarters in New York. The central inquiry revolves around how diplomatic actors use digital tools to complement or augment traditional face-to-face diplomacy.

**Methods:**

We systematically compare a substantial corpus of X posts (tweets) from UN diplomats with their public statements at the United Nations Security Council (UNSC), employing advanced computational social science techniques. This study applies a range of large-scale text analysis methods, including word embedding, topic modeling, and sentiment analysis, to investigate systematic differences between offline and online communication.

**Results:**

Our analysis reveals that, while the essence of diplomacy remains consistent across both domains, there is strategic selectivity in the use of online platforms by diplomats. Online communication emphasizes non-security topics, ceremonial matters, and prominent policy stances, in contrast to the operational issues common in UNSC deliberations. Additionally, online discourse adopts a less confrontational, more public diplomacy-oriented tone, with variations among countries.

**Discussion:**

This study offers one of the first systematic comparisons between offline and online diplomatic messages. It illuminates how diplomats navigate the digital realm to complement traditional roles. The findings indicate that some elements of public diplomacy and nation branding, directed toward a wider audience far beyond the council chamber, have become an integral part of multilateral diplomacy unfolding at the UNSC.

## 1 Introduction

As the world rapidly integrates with cyberspace, those responsible for managing its affairs, including diplomatic actors, find themselves in a state of rapid adaptation to this new reality. For instance, since the late 2000s, the United States State Department has actively engaged in various online platforms such as X (formerly Twitter) and Facebook, allocating increasing human and institutional resources to social media activities as an integral part of its public diplomacy (Zaharna and Rugh, 2012; Bjola and Holmes, 2015). Other countries and organizations, including the United Nations, have followed suit. In the academic domains of international relations and diplomatic studies, this swift expansion of diplomacy into cyberspace has generated growing scholarly interest in the realm of “digital diplomacy” and even “hybrid diplomacy” (Bjola and Holmes, 2015; Bjola and Manor, 2022). These scholars have revealed that diplomatic actors are now actively employing online platforms to gain the public's attention regarding their foreign policy stances and/or improve acceptance among the overseas populace. Despite this burgeoning scholarly attention, however, there is a noticeable lack of research that systematically investigates the conduct of diplomacy spanning *both* the cyber and physical domains. This lack should not be overlooked, as it remains underexplored how the evolving online diplomatic practices function in relation to traditional, dominant, face-to-face practices offline beyond the limited scope of public diplomacy.

The present article addresses this gap, posing the following question: How do diplomatic actors employ digital tools to complement, augment, or even replace traditional face-to-face diplomacy? Exploring this question is vital for understanding the role of digital tools in contemporary diplomacy. To this end, we systematically compare a substantial volume of X posts by diplomats stationed at the United Nations Headquarters in New York with numerous public statements delivered by the same diplomats in traditional face-to-face settings, specifically within the context of policy deliberations at the United Nations Security Council (UNSC). The UNSC, widely regarded as the preeminent multilateral body in global security, provides an ideal case for exploring this question. Its meetings offer opportunities for typical interstate conference diplomacy, with these meetings being characterized by high levels of institutionalization and dominance by major world powers (China, France, Russia, the United States, and the United Kingdom). They consequently stand in stark contrast to the relatively unconstrained realms of cyberspace, open to sovereign states and the general public alike. Our aim is thus to uncover how diplomats, particularly delegates and officials stationed at the UN, leverage this alternative space to conduct their business within the UNSC, an arena historically dominated by face-to-face interactions.

To accomplish this task, we collected and analyzed two distinct sets of text documents under a common methodological framework, fully leveraging advanced computational social science tools. These include over 18,000 speech transcripts extracted from the official meeting records of the UNSC and more than 145,000 X posts by the official X accounts of various countries' permanent missions to the UN and the UN Secretary General. Employing a range of large-scale text analysis methodologies, such as word embedding, topic modeling, and sentiment analysis, we investigate whether systematic differences exist between offline and online communication in terms of semantic content and topic structures. Our analyses reveal that while diplomats do not fundamentally alter the semantic content of their communication—i.e., the meanings they convey when discussing certain concepts—across different domains, they exhibit a high degree of selectivity in their online discourse. In comparison to their offline statements, online posts tend to emphasize non-security matters (e.g., Sustainable Development Goals), ceremonial topics, and policy positions on highly visible issues. Conversely, they generally maintain a more muted stance on operational issues that constitute routine topics in the context of UNSC policy deliberations (e.g., African issues, peacekeeping). Furthermore, the overall tone of online communication is less confrontational and more geared toward public diplomacy and national branding. Notably, significant cross-national variations persist in these and other aspects, underscoring the need for more detailed investigations.

The article is organized as follows: The next section reviews the related literature on digital diplomacy and the UNSC to further illuminate the study's contributions. Section 3 introduces the UNSC speech dataset and the social media posts collected from relevant actors' X accounts. This section also outlines the specific preprocessing and analytical procedures applied to these datasets. Section 4 presents the main findings derived from these procedures (for additional results, see the Supplementary material) and discusses their relevance in the context of existing knowledge on digital diplomacy and council politics. Finally, the last section highlights remaining challenges for this study and suggests promising directions for future research.

## 2 Literature review

Diplomacy is defined as “the conduct of relations between states and other entities with standing in world politics by official agents and by peaceful means” (Bull, 2012, p. 156). In the realm of international relations, diplomacy has historically played a pivotal role in preserving global order. However, the actors involved and the nature of their work have evolved over time. Prior to World War I, foreign ambassadors stationed in host nations held central positions in diplomatic affairs. Post-war, there was a shift toward open diplomacy, driven by the recognition that secret diplomacy had contributed to conflicts. Advances in information transparency led to increased scrutiny of diplomatic activities by legislative bodies and public opinion. Furthermore, diplomacy has transformed, with a rise in multilateral diplomacy and a corresponding decline in bilateral diplomacy, due to the proliferation of international organizations. Politicians and professionals outside of traditional diplomats have consequently become more involved in diplomatic endeavors.

One of the most significant factors influencing the evolution of diplomacy is the development of information and communication technologies (ICTs). The nature of diplomacy has changed in response to rapid communication advancements, such as satellites, airplanes, radios, telegraphs, teletypes, and long-distance telephones (Morgenthau, 1973, p. 536). Important negotiations are now often conducted not only by diplomatic representatives but also by special delegates, including foreign ministers, high officials from foreign offices, or technical experts.

In recent years, ICTs, including social media, have continued to advance, profoundly impacting diplomatic practices. The use of ICTs has prompted European Union permanent representatives to redefine national interests by communicating more frequently with politicians in their capitals, increasing the likelihood of reaching compromises through joint editing of drafts, and emphasizing the importance of language skills in online draft editing (Adler-Nissen and Drieschova, 2019). Diplomats in Geneva have streamlined negotiations through communication via WhatsApp (Cornut et al., 2022).

The rise of social media is of particular note, as it has enabled diplomatic actors to directly engage with citizens and amplified the influence of non-state entities. This shift has given rise to the concept of digital diplomacy, defined as the use of social media for diplomatic purposes (Bjola and Holmes, 2015, p. 4). Notably, the covert use of social media allows state leaders to influence politics through propaganda, advocacy of controversial viewpoints, and the spreading of disinformation (Martin et al., 2023). Non-state actors, including rebels (Bos and Melissen, 2019) and non-governmental organizations (NGOs) (Hall et al., 2020), have also harnessed social media strategies in their diplomatic endeavors.

Digital diplomacy has been practiced most prominently in public diplomacy. Public diplomacy involves diplomatic communication between political entities (ancient kings and modern nation-states) and the public, both foreign and domestic (Huijgh, 2016, p. 437). Diplomatic actors are now actively pursuing such communication on various social media platforms to attract the public's attention and influence their perceptions. One reason for this is that social media elicits emotions and influences diplomatic relations by appealing to national identity through text and images (Duncombe, 2019). Resident diplomats have increasingly embraced public diplomacy by engaging with non-state actors, with social media serving as a facilitating tool that offers a degree of autonomy (Cooper and Cornut, 2019). Moreover, citizens can actively participate in public diplomacy through social networking services (SNS) by posting comments, sharing content, “liking” posts, using hashtags, mentioning others, and participating in online groups (Huang, 2020).

While a growing body of research explores digital diplomacy, most studies focus on how social media has transformed diplomatic practices and mechanisms. As already suggested, however, there is a noticeable lack of research into how digital diplomacy complements or augments traditional face-to-face diplomacy, such as conference diplomacy.

The present study conducts exactly such an investigation. It focuses on permanent representatives at the UNSC and performs a comparative analysis of their speeches in UNSC meetings and their X posts. The analysis aims to illuminate the extent to which permanent representatives use X to complement or emphasize the content of their UNSC speeches.

UNSC meetings represent a typical form of conference diplomacy, defined as multiparty diplomatic negotiations (Meerts, 2016, p. 499). These conferences, often within international organizations, serve as focal points in ongoing negotiation processes and offer relatively stable structures that facilitate successful outcomes (Meerts, 2015, p. 313). While conference diplomacy speeches do involve disseminating information to the public, the structured nature of international organization-based conference diplomacy may dilute the intended message. Consequently, diplomats may seek to use social media to complement and emphasize their conference speeches. The UNSC, being one of the most important conferences in the international arena, offers an ideal case to examine how permanent representatives of international organizations employ (or do not employ) social media to complement and accentuate their conference speeches.

Previous research on public diplomacy using social media has primarily conducted qualitative analyses of public diplomacy content (Ociepka, 2018), explored the network structure of interactive connections among social media users (Huang and Wang, 2021), or assessed its impact on citizens through survey experiments (Min and Luqiu, 2021). For conference diplomacy in the UNSC, diplomatic communication has been analyzed mostly in a qualitative manner, which is typically based on detailed reading of a limited number of resolutions and speeches in UNSC meetings. A few exceptions employing quantitative text analysis include the use of the Latent Dirichlet Allocation method to analyze council resolutions (Hanania, 2021), speaker-topic network analysis of meeting records on Afghanistan (Eckhard et al., 2023), sentiment analysis to assess the validity of the norm of “the responsibility to protect” (Scherzinger, 2023), and word-embedding analysis to explore the meaning of the “threat to the peace” (Sakamoto, 2023b). These quantitative studies, however, have focused narrowly on the textual materials produced in the physical domain, that is, inside the council chamber.

In contrast, this article employs a series of quantitative text analysis tools to compare the speeches delivered by permanent representatives during UNSC meetings with the online messages posted by these same diplomats on X (formerly Twitter). This analytical approach allows for a systematic comparison and examination of content in both physical and digital diplomatic communication, thereby offering a unique opportunity to illuminate the still-unexplored relationship between offline and online diplomatic practices.

## 3 Data and methods

### 3.1 UNSC speeches

We employed the “UNSC Meetings and Speeches” dataset (Sakamoto, 2023a) for our study, which includes English transcripts of all public statements presented by representatives and officials from various countries and organizations during official UNSC meetings.^1^ This dataset covers meetings from 1990 to 2021. For the present study, we updated the dataset by incorporating records from 2022 onward. Specifically, we obtained additional meeting records from the Official Document System of the UN website, accessed via hyperlinks embedded within summary tables presented on the Dag Hammarskjöld Library website.^2^ These English transcripts of statements in public meetings from January 2022 to July 2023 were merged with the existing dataset.

Furthermore, we focused our analysis on meetings held after 2015, the year by which all of the council's five permanent members (P5) had established their respective X/Twitter accounts. Accordingly, our updated dataset comprises public speeches in UNSC meetings from the 7355th session on January 6, 2015, to the 9390th on July 31, 2023, totaling 18,173 statements or 12,259,937 tokens. These statements are from the P5 members, the United Nations Secretary-General (UNSG), and elected representatives, representing a total of 46 distinct entities (Table 1).

**Table 1:** UN speech and X post corpora (2015–2023).

	**Speeches**	**X posts**
Russia	1,539 (1,239,688)	8,752 (3,36,573)
United States	1,364 (1,045,744)	3,991 (112,649)
China	1,283 (763,270)	4,835 (144,518)
France	1,235 (879,177)	8,159 (215,043)
United Kingdom	1,123 (727,361)	10,809 (295,777)
Japan	479 (243,482)	4,045 (127,754)
Brazil	467 (285,009)	1,345 (42,340)
India	442 (275,902)	2,614 (64,598)
Ukraine	405 (313,353)	3,374 (99,791)
Germany	397 (252,036)	6,867 (216,008)
UNSG	146 (146,411)	9,815 (216,544)
All	18,405 (12,367,970)	145,345 (4,162,290)

The total number of speeches (left column) and X posts (right column) delivered/posted by major diplomatic actors, including permanent representatives of the P5 (China, France, Russia, the United States, and the United Kingdom) to the United Nations during the study period (2015–2023). The number in parentheses in each cell indicates the total number of words contained in the corresponding set of speeches/X posts. “UNSG” denotes the UN Secretary-General, whereas “All” refers to the entire set of diplomatic actors under investigation (46 countries and organizations).

### 3.2 UN mission X posts

A further component of the present study involved analyzing posts on X, formerly known as Twitter. Initially limited to 140 characters, the character limit was expanded to 280 starting in 2017.^3^ X serves as a social networking service primarily focused on text communication but also accommodating photographs, images, and videos. Many governments have established UN Permanent Representative accounts on X for public diplomacy purposes. To align our analysis with discussions relevant to the UNSC, we concentrated on accounts associated with UN missions based in New York, excluding accounts related to activities in other locations such as Geneva and Rome.

For the sake of consistency, we excluded personal accounts of ambassadors, focusing solely on the institutional accounts of permanent missions. While we initially considered including the accounts of the UNSG, we excluded them due to personnel turnover, opting instead for the Spokesperson's account as a stable institutional representation. Our analysis covers posts from January 2015 to July 2023, corresponding, as suggested above, to the period during which all P5 countries had held Permanent Representative accounts. Thus, the UN Permanent Representatives accounts included in our analysis are: (1) accounts from P5 countries; (2) accounts from countries that served as non-permanent members of the UNSC from 2015 onward and maintained active accounts during their term; and (3) the account of the Spokesperson for the UNSG. A total of 49 accounts met these criteria (Supplementary Table 1).

For consistency, we excluded posts written in languages other than English and omitted reposts (commonly known as retweets) and quote posts (also known as quote tweets) during data acquisition. Consequently, our analysis covered a total of 145,345 posts, comprising 4,162,290 tokens (Table 1).

### 3.3 Text preprocessing and analyses^4^

We applied a common set of preprocessing measures to both corpora, including lowercase conversion, punctuation removal, and spelling conversion from British to American English. For X posts, we also converted flag emojis, frequently found in social media posts by diplomats, to the names of the corresponding countries and organizations while removing other emojis. Additionally, abbreviations such as “int'l” (for “international”) and “gov't” (for “government”) were expanded.

For analysis purposes, we conducted word counting, word embedding, topic modeling, and sentiment analysis.

#### 3.3.1 Word counting and embedding

To explore variations in the usage of relevant concepts across physical and digital domains, we performed frequency analysis of selected words such as “security,” “peace,” “women,” and “sdg.”^5^ Detailed results are presented in Supplementary Table 2.

We also conducted word embedding for each corpus using the GloVe (Global Vectors for Word Representation) algorithm (Pennington et al., 2014).^6^ Following Sakamoto (2023b), we derived “nearest neighbors”—the words whose embedding vectors are the closest to the vector representing a certain notion—for some of the defining notions in council policymaking as well as in broader international relations. These notions include those represented by words such as “threat,” “protect,” and “sovereignty” (word stem: “sovereignti”). Words were embedded using a common set of learning parameters. In particular, the size (dimensions) of a vector and the size of the context window (in both reading directions) were set to be 100 and 12, respectively. These values were chosen based on the recommendations of prior work (Rodriguez and Spirling, 2021).

#### 3.3.2 Topic modeling

We further examined how different the overall semantic structures are between the offline statements and the online posts. Specifically, we applied Latent Dirichlet Allocation (LDA), which is the simplest and most commonly used Bayesian topic model (Blei et al., 2003), to the combined corpus to estimate the contents of topics—groups of frequently co-occurring words—and their prevalence among the diplomats' messages. We then aggregated the estimated topic prevalence in each text (document-topic vector) for the entire corpus as well as its different subsets such as the speech and X post components. We also derived the country-level semantic structure for each country by simply averaging the estimated document-level topic prevalence across the texts (speeches and X posts) generated by its representatives.

In LDA, the number of topics (denoted as *k*) is exogenously given. We broadly manipulated this parameter (from *k* = 10 to *k* = 60) and iterated estimation ten times for each *k*. The next section details the results obtained from the estimation for *k*=30, whose perplexity—a commonly used metric to evaluate the predictive performance of topic models—was the lowest (indicating the best performance) among the ten iterations. Supplementary Figures 1, 2 display the topic prevalence and its offline-online differences for *k* = 20 and *k* = 50, respectively. While the granularity of the estimated topic structure obviously changes with *k*, the qualitative characterizations of such structure and its offline-online variation given below largely hold for a broad range of *k*.

#### 3.3.3 Sentiment analysis

Finally, we conducted sentiment analysis on both corpora to classify texts as representing “negative,” “positive,” or “neutral” sentiments. We utilized pre-trained sentiment classifiers based on Transformer and its variants (Devlin et al., 2018; Liu et al., 2019). While we did not train classifiers with our labeled data, we manually assessed the performance of various candidate models against hundreds of sample texts from both the speech and X post datasets.^7^ We selected a specific model (pipeline name: lxyuan/distilbert-base-multilingual-cased-sentiments-student) based on its consistency with our manual classification of the samples. The results obtained with an alternative model are presented in Supplementary Figure 3.^8^

We then applied the selected model to the entire body of texts for classification. As most of the speech texts are excessively lengthy for typical sentiment analysis models to handle, we randomly clipped continuous sentences from these texts (subject to a length limit of around 400 characters) and input these “representative” sentences into the model for sentiment classification. Lastly, we aggregated the predicted sentiment of each text over all sets of speeches, the entire set of X posts, and other subsets of the corpora as sentiment distributions for comparison.

## 4 Results

### 4.1 Use of concepts

The word-level analyses reveal that diplomats' usage of different concepts in cyberspace shows no systematic difference from their usage inside the council chamber. In other words, these diplomats are largely consistent in their use of words. As Supplementary Table 2 illustrates, most of the key concepts in contemporary international relations, including “secur(ity),” “peac(e),” “threat,” and “sovereignti (sovereignty),” appear in their offline statements and online posts in more or less comparable frequencies. Several notable exceptions include “SDG” and “global goals” (both denoting Sustainable Development Goals, SDGs), which have been actively promoted online by many UN missions (as well as the UN itself) but are not necessarily favored topics for traditional security organs such as the Security Council.

Word embeddings confirmed the conceptual stability across the cyber and physical domains in a much deeper sense. Diplomats associate a largely similar set of objects and entities with a certain notion, whether they discuss the notion offline or online. Table 2 illustrates this by displaying, for each of the key concepts listed in the first column, its 20 “nearest neighbors”—the top 20 words (stems) whose embedding vectors are most closely located to the vector representing the concept concerned—both offline (second column) and online (third column). For example, the first row indicates that the “threat” notion has been most closely associated with “pose,” “challeng(e),” “terror(ism),” “risk,” “face,” “grow(ing),” and so on when discussed in the council chamber, whereas the same notion has been most strongly associated with “pose,” “challeng(e),” “face,” “risk,” “terror(ism),” “secur(ity),” and so forth when posted online. Notice that the two sets of stems are almost identical to each other in a certain range (the first 10 or so stems in this case). Such extensive overlap tends to be observed as long as the offline and online frequencies of the concept concerned are not excessively divergent from each other (see Supplementary Table 2).

**Table 2:** Semantic associations derived from word embeddings.

**notion**	**neighbors (speeches)**	**neighbors (X posts)**
threat	pose challeng terror risk face grow seriou counter terrorist secur address danger global respond problem tackl complex prolifer confront given	pose challeng face terror risk secur seriou act terrorist requir counter intern address global tackl caus chang impact threaten transnat
peace	achiev sustain stabil process secur last promot reconcili advanc way onli develop believ polit ensur realiz solut inclus effort bring	secur process sustain stabil last build promot achiev effort import toward inclus advanc reconcili solut way intern polit women onli
humanitarian	aid assist need relief dire access deliveri situat crisi worker emerg urgent allow continu call medic provid intern syria civilian	aid assist hum need respons situat access crisi dire urgent syria deliveri crise emerg provid continu call intern relief allow
protect	civilian ensur particular especi must children safeti essenti right secondli respons includ need human women law respect thirdli prevent personnel	children civilian ensur must right promot human need import conflict safeti respect prevent includ essenti commit strengthen safeguard crucial focu
human rights	law fundament violat abuss respect protect defend freedom particular account rule equal includ intern ensur must digniti secondli basic crime	4truth defend fundament violat protect respect abuss equal promot law must freedom person ensur digniti commit women peopl includ intern
sovereignty	territori integr respect sovereign uniti independ principl uphold legitim ownership recogn preserv fundament non-interfer reaffirm neighbor law ukrain charter within	territori integr independ uniti unwav ukraine' sovereign respect recogn within reaffirm reiter ukrain border prevnt'n iraq restor charter principl preserv
democracy	prosper freedom consolid rule fundament restor right democrat path inclus digniti institut govern stabil plural promot toward foundat stabl peac	freedom democrat rule defend fundament prosper promot valu justic right human consolid free path respect restor pursu peac digniti independ

The first column lists significant notions in the context of the UNSC policy deliberations. For each of these notions, the second and third columns in the corresponding row display the 20 “nearest neighbors” for the speech dataset and for the X post dataset, respectively.

### 4.2 Topic structure

**Figure 1:**
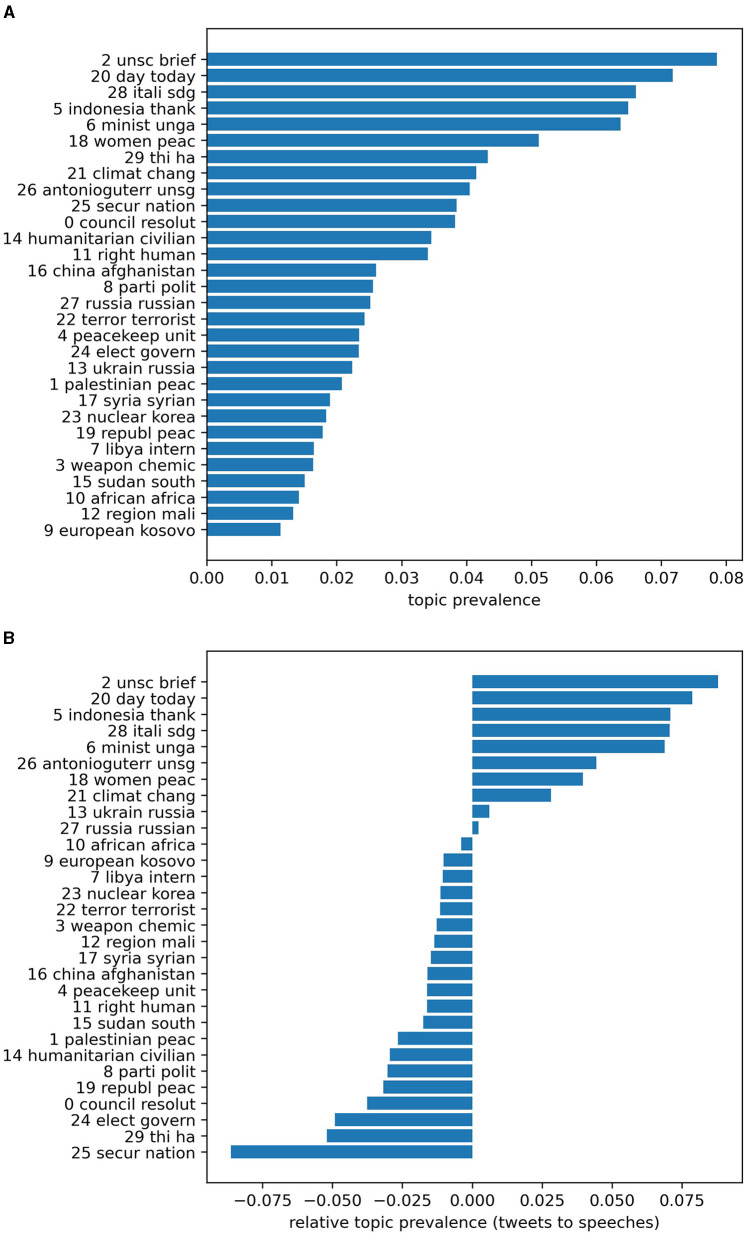
Topic prevalence (*k* = 30). **(A)** Prevalence across the combined corpus; **(B)** Differences in prevalence between the X posts and the speeches.

However, the strong similarity between offline and online communication does not extend further beyond the level of individual concepts. We found noticeable discrepancies between the offline semantic structure and the online structure at the corpus level, although there is also a considerable degree of mutual overlap. For example, Figure 1 illuminates the prevalence of the 30 latent topics—the groups of frequently co-occurring word stems listed in Table 3—that were estimated by the LDA. Panel (A) depicts the overall prevalence of these topics over the entire corpus (the speeches and X posts combined), whereas Panel (B) illustrates the relative topic prevalence in the X posts, obtained by subtracting offline topic weights from those online.

**Table 3:** Estimated topics for the combined corpus (*k* = 30).

**topic**	**associated tokens**
0	council (0.056) resolut (0.052) secur (0.028) adopt (0.021) member (0.021) committe (0.018) work (0.017) sanction (0.013) thi (0.012) implement (0.010)
1	palestinian (0.032) peac (0.018) east (0.017) israel (0.016) solut (0.013) intern (0.013) middl (0.012) isra (0.012) palestin (0.011) gaza (0.009)
2	unsc (0.123) brief (0.038) today (0.034) secur (0.033) live (0.030) meet (0.024) council (0.023) statement (0.020) amb (0.020) watch (0.018)
3	weapon (0.065) chemic (0.035) use (0.032) syria (0.018) opcw (0.015) investig (0.015) convent (0.013) arm (0.011) intern (0.010) non (0.010)
4	peacekeep (0.049) unit (0.034) nation (0.032) mission (0.031) oper (0.030) mandat (0.015) polic (0.013) train (0.011) support (0.011) contribut (0.011)
5	indonesia (0.027) thank (0.022) work (0.018) look (0.016) amb (0.014) forward (0.014) presid (0.012) congratul (0.012) member (0.010) diskuss (0.010)
6	minist (0.033) unga (0.030) switzerland (0.025) unit (0.022) meet (0.020) gener (0.017) nation (0.015) foreign (0.015) kuwait (0.014) state (0.014)
7	libya (0.050) intern (0.031) justic (0.024) libyan (0.023) court (0.020) crime (0.019) mechan (0.016) crimin (0.016) support (0.015) prosecutor (0.015)
8	parti (0.027) polit (0.022) yemen (0.022) effort (0.014) agreement (0.014) solut (0.012) support (0.012) humanitarian (0.011) unit (0.011) special (0.010)
9	european (0.037) kosovo (0.030) union (0.026) eu (0.023) bosnia (0.017) herzegovina (0.016) repres (0.012) high (0.010) agreement (0.010) dialog (0.009)
10	african (0.060) africa (0.055) somalia (0.041) union (0.032) secur (0.028) peac (0.023) support (0.019) au (0.016) ethiopia (0.014) kenya (0.013)
11	right (0.035) human (0.034) conflict (0.029) children (0.029) violenc (0.025) protect (0.021) sexual (0.018) arm (0.016) violat (0.013) account (0.011)
12	region (0.038) mali (0.029) sahel (0.028) secur (0.018) support (0.017) forc (0.015) countri (0.013) west (0.012) guinea (0.011) joint (0.011)
13	ukrain (0.097) russia (0.058) war (0.033) russian (0.020) ukrainian (0.017) aggress (0.015) unit (0.012) intern (0.011) territori (0.011) charter (0.010)
14	humanitarian (0.059) civilian (0.027) peopl (0.017) intern (0.016) million (0.015) need (0.014) assist (0.014) protect (0.011) access (0.011) attack (0.010)
15	sudan (0.057) south (0.046) peac (0.016) darfur (0.015) govern (0.013) agreement (0.012) support (0.011) sudanes (0.010) transit (0.010) implement (0.009)
16	china (0.034) afghanistan (0.032) countri (0.027) intern (0.021) develop (0.020) peac (0.019) region (0.018) commun (0.016) afghan (0.016) support (0.015)
17	syria (0.062) syrian (0.042) humanitarian (0.020) polit (0.016) cross (0.011) regim (0.011) unit (0.011) al (0.010) need (0.009) resolut (0.009)
18	women (0.107) peac (0.026) gender (0.022) particip (0.021) youth (0.018) societi (0.016) girl (0.016) equal (0.016) right (0.012) young (0.012)
19	republ (0.032) peac (0.023) african (0.019) central (0.018) democrat (0.017) congo (0.016) region (0.015) colombia (0.013) support (0.011) countri (0.010)
20	day (0.026) today (0.020) world (0.018) celebr (0.015) year (0.014) join (0.013) thi (0.011) live (0.011) malta (0.010) event (0.010)
21	climat (0.036) chang (0.024) global (0.022) food (0.014) water (0.013) pandem (0.013) countri (0.012) need (0.012) challeng (0.012) develop (0.012)
22	terror (0.040) terrorist (0.036) counter (0.021) state (0.015) threat (0.015) group (0.013) intern (0.011) iraq (0.011) combat (0.009) fight (0.008)
23	nuclear (0.041) korea (0.020) iran (0.018) secur (0.014) republ (0.014) state (0.013) democrat (0.012) missil (0.012) intern (0.012) peopl (0.011)
24	elect (0.019) govern (0.018) nation (0.018) support (0.016) polit (0.015) unit (0.013) haiti (0.012) process (0.011) repres (0.010) welcom (0.010)
25	secur (0.024) nation (0.023) peac (0.022) conflict (0.019) unit (0.017) intern (0.016) council (0.013) prevent (0.011) develop (0.010) organ (0.010)
26	antonioguterr (0.051) unsg (0.038) ban (0.035) moon (0.030) ki (0.030) norway (0.024) condemn (0.023) attack (0.022) secretary (0.018) gener (0.018)
27	russia (0.026) russian (0.021) ukrain (0.016) state (0.012) nebenzia (0.011) wa (0.010) osc (0.010) ukrainian (0.009) militari (0.009) forc (0.008)
28	itali (0.024) sdg (0.021) develop (0.018) support (0.013) sustain (0.013) commit (0.010) achiev (0.009) amb (0.009) tribun (0.008) work (0.007)
29	thi (0.023) ha (0.022) unit (0.016) council (0.016) wa (0.013) peopl (0.012) today (0.010) time (0.010) veri (0.009) year (0.009)

The 10 tokens listed in each row are the word stems most strongly associated with the corresponding topic. A number in parentheses denotes the strength (weight) of association.

A quick comparison of the two graphs reveals that many of the topics that dominate the entire corpus such as topics 2, 20, 28, 5, and 6 have been disproportionately mentioned in the cyber domain. These topics represent the announcement of an upcoming council meeting or a representative's diplomatic statement there (2); daily reminders of a ceremonial event or an anniversary (20); promotion of SDGs (28); a greeting to a fellow member state relating to its work in the UNSC or in the wider UN (5); and information on an event regarding the UN General Assembly (mentioned by “unga”) rather than UNSC (6) (see Table 3). Other topics, including those relating to global issues such as climate change and the pandemic (21), the “Women, Peace, Security (WPS)” agenda (18), and the activities of the UN Secretary-General, or the UNSG (26), also tend to be actively mentioned online.

In contrast, there are also topics that have been disproportionately discussed in the physical domain. These topics concern the UNSC's role in preventing conflict and building sustainable peace (25) as well as its engagement with political processes and elections in places such as Haiti (24). Meanwhile, topic 29 comprises a pool of frequent terms and established expressions in the specific context of the UNSC deliberations. Other topics, including humanitarian aid and protection of civilians in conflict (14), peacekeeping operations (4), the Israeli-Palestinian conflict (1), and other specific conflict situations (19, 8, 15, 16, 17, 12), also show a similar inclination to be discussed more intensely in the council chamber, albeit to a lesser extent.

Notably, some topics, including those concerning Russian aggression against Ukraine (13, 27), have been attracting the active engagement of UN-based diplomats in both domains, so their prevalence is somehow balanced between the speech and X post corpora. These topics constitute a limited area of overlap between the offline and online semantic structures, which are otherwise largely distinct.

As mentioned earlier, the estimations of the LDA and other topic models depend on several factors, among others, the number of topics ‘k' and the random seed used for initialization. Therefore, the exact compositions of estimated topics and their quantitative distributions in the speeches and the X posts can vary considerably depending on these conditions. Nevertheless, the overall patterns of semantic divergence and convergence described above hold in a broad range of parameters and iterations, at least qualitatively (see Supplementary Figures 1, 2). These patterns are also partially supported by the aforementioned word frequency analysis. As Supplementary Table 2 displays, some words such as “sdg,” “women,” and “gender” appear distinctly more frequently in the X posts than in the speeches, which is largely consistent with the relative prevalence of the topics these words obviously relate to. Similarly, other words that constitute distinctively “offline topics” for council deliberations—for example, the names of some African countries such as “congo” and “south sudan” (but not “somalia” and “mali”)—appear far more frequently in offline statements than in online posts.

These results suggest a considerable degree of selectivity regarding what diplomatic actors talk about when they are online or offline. Inside the council chamber, delegates typically focus on practical security matters that, having been a part of regular meeting agendas for many years, have become almost routine in the context of council politics. These matters concern protracted conflict situations in the Middle East and Africa (topics 1, 8, 15, 19, etc.), and the UN's involvement with these situations through its peacekeeping operations and other endeavors (4, 14, 24, 25). In contrast, the same diplomats appear to focus on more general, broadly discussed topics such as SDGs, climate change, and WPS (5, 18, 28) when they post on X. This clear divergence in the semantic structure of diplomatic communication indicates that the Security Council, by its nature as a conference diplomacy, tends to direct diplomats' attention to narrowly conceived security matters, whereas X, which has a high degree of freedom, may be utilized to transmit more generalized information to a much wider audience.

It should be noted, however, that some of the concrete and traditional security issues typically discussed in the council chamber are also actively mentioned on X. The heatedly debated war between Russia and Ukraine (topics 13, 27) is a prime example. To a much lesser extent, concerns related to weapons of mass destruction—specifically, the use of chemical weapons in Syria (3) and North Korea and Iran's nuclear programs (23)—and global terrorism (22) are intensely discussed on the online platform, not just in offline council meetings. These topics appear to be more visible to the general public, especially in the United States and Europe, in comparison with other topics such as conflicts in Africa and the Middle East, which have been disproportionately discussed in the council chamber. These patterns suggest that diplomats might strategically differentiate between security topics best suited for offline, conference-based channels and those more appropriate for online, public channels, according to the target of diplomacy.

### 4.3 Tone

**Figure 2:**
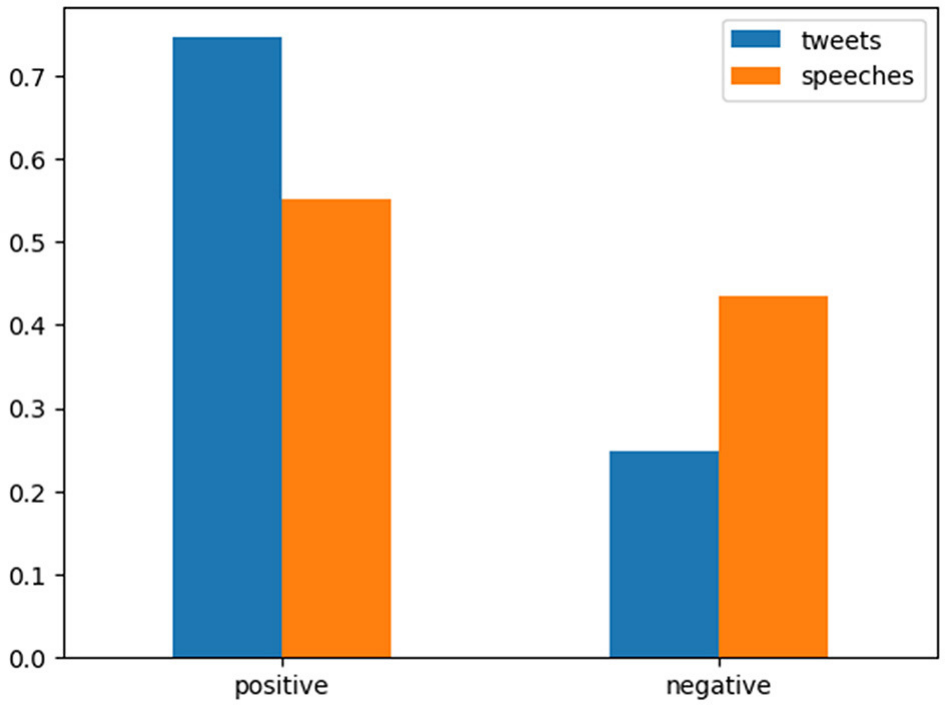
Offline and online sentiment distributions. The X posts/speeches classified as “neutral,” which constitute a negligible portion, were excluded from the figure for readability.

With regard to the document-level sentiment analysis, we found a striking difference between the two corpora. As Figure 2 demonstrates, in comparison with the offline policy statements, the online diplomatic posts express less negative and more positive sentiments. In other words, it seems that UN-based diplomats are less confrontational in cyberspace than in the physical domain. This general tendency for a more positive tone in social media was supported by a similar analysis using an alternative sentiment classifier (see Supplementary Figure 3), even though the exact distributions of positive, neutral, and negative sentiments predicted by different models are considerably divergent. The tendency was also consistent with our unambiguous impression gained from a manual reading of X posts and speeches. That is, compared to often-heated exchanges in the council chamber, the X posts by diplomats noticeably contain advertising, forward-looking, and even highly casual expressions.

These features might seem somewhat surprising given the widely held belief that social-media exchanges likely induce polarization and conflict (Martin et al., 2023). However, they can be interpreted through nation branding in a rather straightforward manner. In the realm of public diplomacy, the concept of nation branding is a subject of considerable discussion. Nation branding is “a process by which a nation's images can be created or altered, monitored, evaluated and proactively managed in order to enhance the country's reputation among a target international audience” (Fan, 2010, p. 101). Given that X serves as a tool for public engagement, it is reasonable to hypothesize that nation-branding initiatives may be operational through this channel. It is likewise conceivable that a strategy might be in place to limit negative expressions and highlight positive ones, with the aim of creating a more favorable impression of one's country. Two X posts classified as positive are provided below as an illustration of such national-branding strategy.

“Through its membership at #ECOSOC, Indonesia is fully committed to actively contribute to promoting transformative actions to accelerate the implementation of SDGs. #inidiplomasi” (Indonesian Mission UN, 2023).

“The United States is deeply committed to preventing and responding to human trafficking around the world. We stand in solidarity with all those around the world working to” #EndHumanTrafficking (U.S. Mission to the UN, 2023).

### 4.4 Actor-level analyses

**Figure 3:**
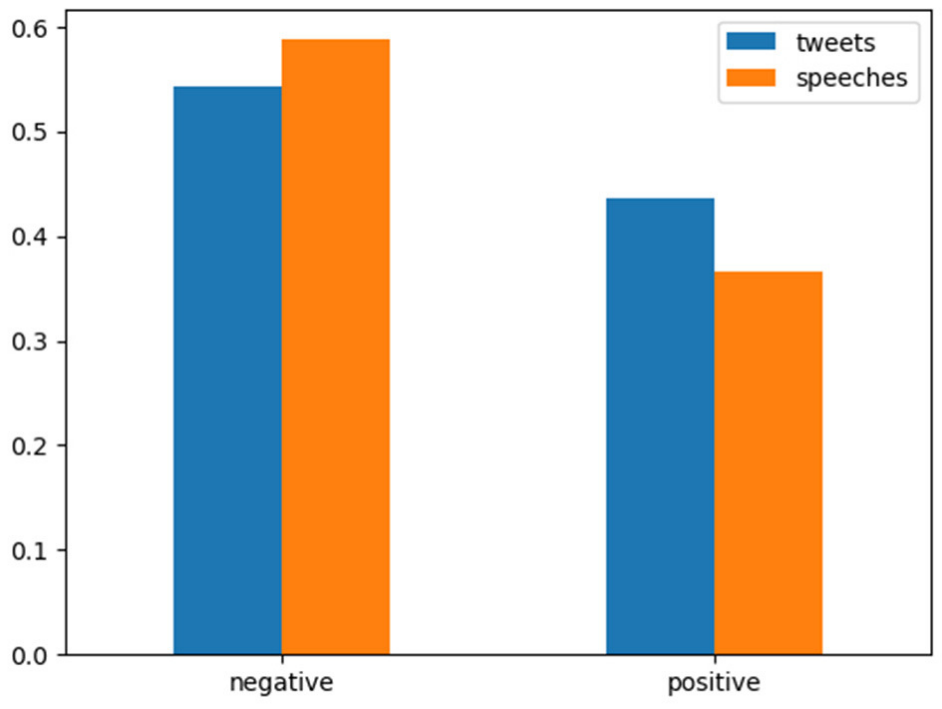
Offline and online sentiment distributions in the case of Russia. The X posts/speeches classified as “neutral” were excluded from the figure for readability.

We also conducted a preliminary investigation into how the offline-online differences depicted above can vary across different UN missions, especially among dominant permanent members (P5). Although the investigation was far from exhaustive, we gained an indication that there is indeed a considerable degree of cross-national variation concerning the corpus-level semantic structure as well as the distribution of document-level sentiment tones. As a conspicuous example, Figure 3 depicts the sentiment distributions for the offline statements and the online posts by the Russian mission at the UN. A casual comparison with Figure 2 quickly reveals the markedly confrontational posture of Russian diplomats regardless of offline or online status. This posture stands in marked contrast to that of the other permanent members, including China (Supplementary Figure 4) and the U.S. (Supplementary Figure 5). In particular, the sentiment distributions for the Chinese mission indicate the relatively restrained nature of the country's diplomatic messages.

Lastly, Supplementary Figure 6 illustrates another dimension of cross-national difference, namely the relative topic prevalence. Each graph was generated from country-level aggregation of topic prevalence over the X posts and the speeches for *k* = 30 (Table 3). While the configurations of predominantly offline topics and predominantly online topics are largely similar to that observed for the entire corpus (see Figure 1B), each country features its own emphasis on what it chooses to discuss. For example, in comparison with the other members, China has devoted more attention to topic 16 in the council chamber, emphasizing the country's contribution to peace, development, and stability in Afghanistan and the surrounding region. While the U.S. mission has not shown a particularly distinct tendency in this regard, Russia is again notable in that its online messages disproportionately draw on topic 27. This topic largely represents Russia's efforts to justify its policy, and subsequent war, against Ukraine in terms of what Russia sees as the Western attempt to militarize Ukraine.

These results indicate significant differences among countries in terms of the channels they use to disseminate information, with such variation potentially reflecting differences in each country's nation-branding strategy. However, our analysis of cross-national dimensions remains preliminary, and more extensive investigation is required to draw any definite conclusion in this regard.

## 5 Conclusions

Through the rigorous application of advanced computational social science tools, this study offers one of the first systematic comparisons between offline and online diplomatic messages in the context of the UNSC. These comparisons have uncovered how burgeoning digital diplomacy functionally augments established conference diplomacy. While diplomats maintain consistency in their use of terms and concepts, digital platforms have afforded them an expanded toolkit, enabling more versatile expressions of diplomatic strategies. Inside the council, diplomats engage with policy deliberations on a wide range of security issues in a tightly constrained institutional setting. Conversely, in cyberspace, they can be more selective in what they talk about and more open in how they convey their messages. Specifically, online messages typically emphasize ceremonial topics as well as prominent policy issues, including those not strictly conceived as security matters (e.g., SDGs), rather than other operational issues common in UNSC deliberations. Additionally, online communication adopts a less confrontational, more forward-looking tone, albeit with a considerable degree of cross-national variation. These observations indicate that some elements of public diplomacy and nation branding, directed toward a wider audience far beyond the council chamber, have become an integral part of multilateral diplomacy unfolding at the UNSC.

There are important limitations and remaining challenges following our study. First, it is essential to acknowledge the need for a stricter comparison given the non-identical nature of our two datasets. While the Security Council is one of the most prominent principal organs of the United Nations, the former is nevertheless a part of the latter. Therefore, the discursive range of X posts by UN-based diplomats might inherently be broader than that of their public statements at UNSC meetings, potentially rendering our comparison somewhat unbalanced. Extensive investigation of additional text data from other UN organs, most notably the General Assembly, would likely address this imbalance.^9^

Second, the study is constrained by the preliminary nature of the actor-level analyses and the absence of exhaustive comparisons among countries. This necessitates further research to discern nuanced divergences in diplomatic strategies among different actors. The highlighted disparities in the deployment of topics and tones across platforms and actors advocate for more granular analyses to comprehend the underlying motivations, strategies, and implications of these variances in digital diplomacy.

Third, in addition to more extensive actor-level analysis, there is a need for analysis that is oriented toward the receiving side of diplomatic efforts.^10^ For example, there might be systematic differences between diplomatic messages that attract strong public attention (as measured by the numbers of “likes,” “reposts,” and so on) and those that do not. Preliminary analyses indicate that there is indeed such heterogeneity in diplomatic communication. Specifically, while most of the X posts analyzed here have not aroused any strong public reaction, a small number of posts that have amassed a large number of “likes” tend to mention highly contentious issues such as Ukraine, Palestine, and Myanmar in a considerably assertive, even confrontational, manner. This result is instructive because it indicates that diplomatic actors might not employ digital diplomacy solely for national branding. We will further pursue this line of analysis in future work.

Finally, the methodological approaches employed in this study can be fruitfully utilized in more depth. For example, following preceding work on crisis decision making (e.g., Gibson, 2011), these approaches can be applied to online and offline messages on specific policy issues (e.g., climate change, COVID-19, the Russian invasion against Ukraine, the Israel-Palestine conflict, etc.) to examine how different actors at the UNSC, especially the P5, converge or diverge in their views and policy stances. Such analysis might offer useful insights regarding how multilateral conference diplomacy at the UNSC, the effectiveness of which has often been called into question, can adequately function in the face of serious global threats.

## Data availability statement

The datasets presented in this study can be found in online repositories. The names of the repository/repositories and accession number(s) can be found at: Harvard Dataverse (https://doi.org/10.7910/DVN/CKPTRB).

## Ethics statement

Ethical approval was not required for the study involving human data in accordance with the local legislation and institutional requirements. The social media data was accessed and analyzed in accordance with the platforms' terms of use and all relevant institutional/national regulations.

## Author contributions

TS: Conceptualization, Data curation, Formal analysis, Methodology, Project administration, Writing – original draft, Writing – review & editing. MA: Data curation, Formal analysis, Writing – original draft, Writing – review & editing. HI: Conceptualization, Resources, Writing – original draft, Writing – review & editing. TM: Data curation, Writing – original draft, Writing – review & editing.

## Footnotes

^1^Procedural statements given by the president of each meeting are removed from the data (Sakamoto, 2023b, p. 7).

^2^http://research.un.org/en/docs/sc/quick/meetings/ (last accessed on September 25, 2023).

^3^In 2023, the character limit was extended to 4,000 characters for users subscribed to the paid service “Twitter Blue.”

^4^Unless mentioned otherwise, the preprocessing and analytical measures described in this section were implemented using Python (ver. 3.10) and its various extensions (e.g., Gensim, NLTK).

^5^To be more precise, we counted word stems (e.g., “peac,” “secur”) rather than words themselves (“security,” “peace”).

^6^We used the GloVe implementation code that is available from the developers' website (https://nlp.stanford.edu/projects/glove/; last accessed on September 24, 2023).

^7^These models are available from the Hugging Face platform (https://huggingface.co.; last accessed on September 25, 2023).

^8^See https://huggingface.co/cardiffnlp/twitter-roberta-base-sentiment-latest. (last accessed on September 26, 2023).

^9^Although the General Assembly and its First Committee (focused on disarmament and security issues) maintain verbatim records, as does the Security Council, records from other primary UN bodies such as the Economic and Social Council are not verbatim. Instead, they are summary records, which present challenges for comparative analysis.

^10^We thank a reviewer for suggesting this line of analysis.

## References

[B1] Adler-Nissen R. Drieschova A. (2019). Track-change diplomacy: technology, affordances, and the practice of international negotiations. Int. Stud. Q. 63, 531–545. 10.1093/isq/sqz030

[B2] Bjola C. Holmes M. (2015). Digital diplomacy: Theory and practice. Abingdon: Taylor and Francis.

[B3] Bjola C. Manor I. (2022). The rise of hybrid diplomacy: from digital adaptation to digital adoption. Int. Aff. 98, 471–491. 10.1093/ia/iiac005

[B4] Blei D. M. Ng A. Y. Jordan M. I. (2003). Latent dirichlet allocation. J. Mach. Learn. Res. 3, 993–1022.

[B5] Bos M. Melissen J. (2019). Rebel diplomacy and digital communication: public diplomacy in the Sahel. Int. Aff. 95, 1331–1348. 10.1093/ia/iiz195

[B6] Bull H. (2012). The Anarchical Society: A Study of Order in World Politics, 4th Edn. London: Palgrave Macmillan.

[B7] Cooper A. F. Cornut J. (2019). The changing practices of frontline diplomacy: new directions for inquiry. Rev. Int. Stud. 45, 300–319. 10.1017/S0260210518000505

[B8] Cornut J. Manor I. Blumenthal C. (2022). WhatsApp with diplomatic practices in Geneva? Diplomats, digital technologies, and adaptation in practice. Int. Stud. Rev. 24:viac047. 10.1093/isr/viac047

[B9] Devlin J. Chang M.-W. Lee K. Toutanova K. (2018). BERT: pre-training of deep bidirectional transformers for language understanding. arXiv [Preprint]. arXiv: 1810.04805.

[B10] Duncombe C. (2019). Digital diplomacy: emotion and identity in the public realm. Hague J. Dipl. 14, 102–116. 10.1163/1871191X-14101016

[B11] Eckhard S. Patz R. Schönfeld M. van Meegdenburg H. (2023). International bureaucrats in the UN security council debates: a speaker-topic network analysis. J. Eur. Public Policy 30, 214–233. 10.1080/13501763.2021.1998194

[B12] Fan Y. (2010). Branding the nation: towards a better understanding. Place Brand. Public Dipl. 6, 97–103. 10.1057/pb.2010.16

[B13] Gibson D. R. (2011). Avoiding catastrophe: the interactional production of possibility during the cuban missile crisis. Am. J. Soc. 117, 361–419. 10.1086/661761

[B14] Hall N. Schmitz H. P. Dedmon J. M. (2020). Transnational advocacy and NGOs in the digital era: New forms of networked power. Int. Stud. Q. 64, 159–167. 10.1093/isq/sqz052

[B15] Hanania R. (2021). The Humanitarian turn at the UNSC: explaining the development of international norms through machine learning algorithms. J. Peace Res. 58, 655–670. 10.1177/0022343320929728

[B16] Huang Q. E. (2020). Facebook not statebook: defining SNS diplomacy with four modes of online diplomatic participation. Int. J. Commun. 14, 3885–3902.

[B17] Huang Z. A. Wang R. (2021). Exploring China's digitalization of public diplomacy on Weibo and Twitter: a case study of the US–China trade war. Int. J. Commun. 15, 1912–1939.

[B18] Huijgh E. (2016). “Public diplomacy”, in The SAGE Handbook of Diplomacy, eds. C.M. Constantinou, P. Kerr, and P. Sharp (London: SAGE Publications), 437–450.

[B19] Indonesian Mission UN (2023). Available online at: https://x.com/indonesiaunny/status/1684611052229492747 (accessed September 29, 2023).

[B20] Liu Y. Ott M. Goyal N. Du J. Joshi M. Chen D.. (2019). RoBERTa: a robustly optimized BERT pretraining approach. arXiv [Preprint]. arXiv: 1907.11692.

[B21] Martin D. A. Shapiro J. N. Ilhardt J. G. (2023). Introducing the online political influence efforts dataset. J. Peace Res. 60, 868–876. 10.1177/00223433221092815

[B22] Meerts P. (2015). Diplomatic Negotiation: Essence and Evolution. The Hague: Clingendael Institute.

[B23] Meerts P. (2016). “Conference diplomacy,” in The SAGE Handbook of Diplomacy, eds. C.M. Constantinou, P. Kerr, and P. Sharp (London: SAGE Publications), 499–509.

[B24] Min B. Luqiu L. R. (2021). How propaganda techniques leverage their advantages: a cross-national study of the effects of Chinese international propaganda on the US and South Korean audiences. Polit Commun. 38, 305–325. 10.1080/10584609.2020.1763524

[B25] Morgenthau H. (1973). Politics Among Nations: The Struggle for Power and Peace, 5th Edn. New York, NY: Alfred A. Knopf.

[B26] Ociepka B. (2018). Public diplomacy as political communication: lessons from case studies. Eur. J. Commun. 33, 290–303. 10.1177/0267323118763909

[B27] Pennington J. Socher R. Manning C. (2014). “GloVe: global vectors for word representation,” in Proceedings of the 2014 Conference on Empirical Methods in Natural Language Processing (EMNLP), 1532–1543. 10.3115/v1/D14-1162

[B28] Rodriguez P. L. Spirling A. (2021). Word embeddings: what works, what doesn't, and how to tell the difference for applied research. J. Politics 84, 101–115. 10.1086/715162

[B29] Sakamoto T. (2023a). The UNSC Meetings and Speeches, Harvard Dataverse. 10.7910/DVN/CKPTRB

[B30] Sakamoto T. (2023b). Threat conceptions in global security discourse: analyzing the speech records of the United Nations security council, 1990–2019. Int. Stud. Q. 67, sqad067. 10.1093/isq/sqad067

[B31] Scherzinger J. (2023). Unbowed, unbent, unbroken? Examining the validity of the responsibility to protect. Coop. Confl. 58, 81–101. 10.1177/00108367221093155

[B32] U.S. Mission to the UN (2023). Available online at: https://x.com/USUN/status/1685760623685021698 (accessed September 29, 2023).

[B33] Zaharna R. S. Rugh W. A. (2012). The use of social media in U.S. public diplomacy. Glob. Media J. 12, 1–8.

