# Supplementary material for: How different are offline and online diplomacy? A comparative analysis of public statements and SNS posts by delegates to the United Nations
Source: Front Big Data. 2024 Apr 8;7:1304806. doi: 10.3389/fdata.2024.1304806 (PMC11049423; doi:10.3389/fdata.2024.1304806)
Supplement: Supplementary file 1 [file Data_Sheet_1.pdf]

Supplementary Material

1 Supplementary Tables

Supplementary Table 1. X (Twitter) accounts of UN missions

account name	ID	country/organization
Albania in UN	@AlMissionUN	Albania
Belgium UN New York	@BelgiumUN	Belgium
Misión Permanente de Bolivia en la ONU- Nueva York	@BoliviaUNNY	Bolivia
Brazil Mission to the UN	@Brazil_UN_NY	Brazil
ChileONU	@ChileONU	Chile
Chinese Mission to UN	@Chinamission2un	China
Spokesperson of Chinese Mission to UN	@CHN_UN_NY	China
La Côte d’Ivoire à l’ONU	@CotedivoireOnu	Cote d' Ivoire
Dominican Republic Mission to the UNSC Archived	@DominicanRepUN	Dominican Republic
Ecuador en ONU	@EcuadorONU	Ecuador
Estonia in UN | #StandWithUkraine	@EstoniaUN	Estonia
Ethiopia at the UN	@Ethiopia_UN	Ethiopia
La France à l'ONU	@franceonu	France
Germany in the United Nations	@GERMANYonUN	Germany
Germany in the United Nations	@GermanyUN	Germany
GhanaUN	@ghanamissionun	Ghana
India at UN, NY	@IndiaUNNewYork	India
Indonesian Mission UN	@indonesiaunny	Indonesia
Ireland at UN	@irishmissionun	Ireland
Italy UN New York	@ItalyUN_NY	Italy
Japanese Mission UN	@JapanMissionUN	Japan
Mission of Jordan to UN	@JordanUN_NY	Jordan
Kazakhstan Mission at the UN	@KazakhstanUN	Kazakhstan
Permanent Mission of Kenya to the UN	@KenyaMissionUN	Kenya

Supplementary Material

Kuwait Mission to the UN	@KuwaitMissionUN	Kuwait
LithuaniaUN | #StandWithUkraine	@LithuaniaUNNY	Lithuania
Malta at the UN	@MaltaUNMission	Malta
Misión de México ONU	@MexOnu	Mexico
Mission Permanente du Gabon à l'ONU - New York	@MPNY_GabonOnu	Gabon
NIGER MISSION TO THE UN	@Niger_ONU	Niger
Netherlands at UN	@NLatUN	Netherlands
NorwayUN	@NorwayUN	Norway
NZ at the UN	@NZUN	New Zealand
Perú en la ONU	@PeruEnLaONU	Peru
Poland in the UN	@PLinUN	Poland
Tunisia PM to the UN	@PmTunisia	Tunisia
Dominican Republic Mission to the UN	@RDenONU	Dominican Republic
Russia at the United Nations	@RussiaUN	Russian Federation
South Africa at the UN	@SAMissionNY	South Africa
España en la ONU	@SpainUN	Spain
Permanent Mission of SVG to the UN	@SVG_UN	Saint Vincent and the Grenadines
SwedenUN	@SwedenUN	Sweden
SwitzerlandUN	@swiss_un	Switzerland
UAE Mission to the UN	@UAEMissionToUN	United Arab Emirates
UKR Mission to the UN	@UKRinUN	Ukraine
UK at the UN	@UKUN_NewYork	United Kingdom
UN Spokesperson	@UN_Spokesperson	United Nations
U.S. Mission to the UN	@USUN	United States
Viet Nam at UN (NY)	@VietNam_UN	Viet Nam

Supplementary Table 2. Frequencies (per 1000 words) of selected word stems in speeches and X posts

word stem	speeches	tweets	word stem	speeches	tweets
secur	5.470	4.534	democraci	0.102	0.163
peac	4.420	3.870	veto	0.074	0.160
humanitarian	2.662	2.109	global goal	0.001	0.006
women	1.880	2.703	syria	1.084	1.947
children	0.962	1.000	ukrain	1.012	1.868
protect	1.240	1.567	russia	0.859	1.645
human	1.351	1.469	africa	0.679	0.783
peacekeep	1.006	0.980	afghanistan	0.653	0.721
sdg	0.010	1.033	yemen	0.441	0.789
law	1.410	0.835	nuclear	0.549	0.542
climat	0.427	0.965	iraq	0.468	0.369
human right	0.933	0.951	south sudan	0.447	0.171
gender	0.333	0.723	congo	0.415	0.118
terror	0.977	0.714	mali	0.387	0.407
prolifer	0.297	0.183	somalia	0.375	0.430
threat	0.853	0.587	israel	0.350	0.293
war	0.736	0.583	haiti	0.344	0.272
justic	0.580	0.536	iran	0.275	0.237
sanction	0.574	0.517	palestin	0.222	0.230
aggress	0.170	0.229	nato	0.053	0.060
sovereignti	0.264	0.168	global south	0.007	0.015

Supplementary Table 3. Estimated topics for the combined corpus (*k*=20)

topic	associated tokens
0	women(0.083) peac(0.028) violenc(0.022) gender(0.018) sexual(0.017) conflict(0.017) particip(0.016) societi(0.014) girl(0.013) secur(0.011)
1	brief(0.046) live(0.044) today(0.035) antonioguterr(0.034) unsc(0.031) watch(0.028) secur(0.024) council(0.022) meet(0.020) malta(0.017)
2	ukrain(0.049) russia(0.038) russian(0.018) war(0.014) ukrainian(0.013) ha(0.009) territori(0.008) aggress(0.007) militari(0.007) unit(0.007)
3	peac(0.016) govern(0.014) support(0.014) polit(0.012) agreement(0.012) process(0.012) sudan(0.012) nation(0.010) elect(0.009) continu(0.009)
4	palestinian(0.027) peac(0.017) east(0.015) israel(0.014) intern(0.012) solut(0.012) middl(0.011) isra(0.010) secur(0.010) palestin(0.009)
5	unsc(0.108) statement(0.032) amb(0.024) ireland(0.019) today(0.018) adopt(0.018) resolut(0.017) norway(0.016) debat(0.015) meet(0.012)
6	secur(0.023) intern(0.020) peac(0.019) nation(0.015) develop(0.015) countri(0.014) unit(0.011) conflict(0.011) afghanistan(0.011) china(0.010)
7	nation(0.031) unit(0.030) peacekeep(0.023) oper(0.015) secur(0.015) mission(0.015) council(0.015) peac(0.009) support(0.009) mandat(0.009)
8	weapon(0.023) committe(0.020) resolut(0.019) nuclear(0.017) implement(0.015) state(0.013) non(0.012) kosovo(0.011) prolifer(0.010) iran(0.010)
9	presid(0.022) unit(0.021) minist(0.019) meet(0.017) nation(0.016) work(0.015) member(0.014) new(0.013) state(0.011) foreign(0.010)
10	terror(0.034) terrorist(0.031) iraq(0.017) counter(0.017) attack(0.013) state(0.012) group(0.012) threat(0.012) intern(0.009) mali(0.009)
11	humanitarian(0.042) syria(0.016) peopl(0.015) need(0.013) syrian(0.012) civilian(0.011) assist(0.011) million(0.009) situat(0.009) unit(0.008)
12	libya(0.027) polit(0.024) yemen(0.023) parti(0.019) support(0.014) libyan(0.013) effort(0.012) process(0.011) special(0.011) unit(0.011)
13	council(0.025) thi(0.016) ha(0.014) wa(0.012) use(0.011) unit(0.010) state(0.009) resolut(0.009) secur(0.009) weapon(0.008)
14	right(0.043) human(0.040) children(0.038) protect(0.038) conflict(0.034) arm(0.023) civilian(0.016) violat(0.014) law(0.012) intern(0.012)
15	intern(0.031) crime(0.025) justic(0.022) account(0.017) law(0.016) court(0.014) mechan(0.012) investig(0.012) crimin(0.012) state(0.011)
16	itali(0.018) develop(0.015) global(0.014) indonesia(0.013) support(0.013) sdg(0.011) sustain(0.010) covid19(0.009) commit(0.008) need(0.007)
17	somalia(0.034) korea(0.025) secur(0.025) council(0.024) sanction(0.023) peopl(0.022) japan(0.022) republ(0.020) democrat(0.018) resolut(0.016)
18	day(0.017) world(0.016) today(0.014) ban(0.012) thi(0.011) moon(0.011) event(0.011) ki(0.011) celebr(0.010) unsg(0.010)
19	african(0.036) region(0.034) republ(0.020) africa(0.019) secur(0.016) union(0.016) central(0.015) support(0.014) countri(0.012) peac(0.012)

Note: The 10 tokens listed in each row are the word stems most strongly associated with the corresponding topic. A number in parentheses denotes the strength (weight) of association.

Supplementary Table 4. Estimated topics for the combined corpus (*k*=50)

topic	associated tokens
0	minist(0.086) gener(0.073) antonioguterr(0.062) foreign(0.042) secretary(0.033) meet(0.031) unga(0.028) fm(0.023) met(0.021) assembl(0.021)
1	look(0.027) work(0.027) diskuss(0.027) forward(0.025) session(0.017) thank(0.016) meet(0.016) nation(0.015) today(0.014) unit(0.012)
2	council(0.047) secur(0.022) thi(0.016) gener(0.014) like(0.013) resolut(0.013) work(0.012) member(0.012) presid(0.011) issu(0.010)
3	china(0.062) develop(0.060) japan(0.038) countri(0.032) cooper(0.028) water(0.020) econom(0.018) ha(0.014) promot(0.012) build(0.012)
4	thi(0.032) ha(0.024) unit(0.019) council(0.018) peopl(0.013) today(0.012) time(0.012) wa(0.012) veri(0.012) make(0.011)
5	germani(0.084) poland(0.072) amb(0.051) unsc(0.050) int(0.039) mexico(0.036) mtg(0.025) incl(0.025) peacebuild(0.015) support(0.013)
6	indonesia(0.065) covid19(0.037) pandem(0.034) malta(0.029) 19(0.026) covid(0.024) inidiplomasi(0.023) global(0.023) vaccin(0.021) health(0.020)
7	republ(0.078) democrat(0.049) congo(0.044) region(0.029) burundi(0.017) monusco(0.016) group(0.014) arm(0.013) stabil(0.012) great(0.012)
8	resolut(0.134) adopt(0.084) council(0.040) vote(0.040) draft(0.035) mandat(0.032) renew(0.026) unsc(0.025) unanim(0.023) secur(0.023)
9	conflict(0.028) peac(0.028) nation(0.024) prevent(0.017) unit(0.017) develop(0.016) secur(0.016) sustain(0.014) peacebuild(0.011) import(0.011)
10	region(0.053) african(0.049) africa(0.044) sahel(0.024) secur(0.023) union(0.019) support(0.018) countri(0.013) joint(0.012) state(0.012)
11	justic(0.033) crime(0.033) intern(0.031) court(0.024) mechan(0.019) crimin(0.019) account(0.017) prosecutor(0.015) investig(0.012) icc(0.012)
12	cultur(0.032) lebanon(0.020) heritag(0.016) visit(0.014) italian(0.011) unesco(0.010) languag(0.010) exhibit(0.010) usambun(0.009) unifil(0.009)
13	iraq(0.092) iraqi(0.028) govern(0.026) da(0.023) esh(0.022) support(0.022) team(0.019) isil(0.015) commit(0.014) welcom(0.013)
14	weapon(0.062) chemic(0.054) use(0.042) syria(0.030) opcw(0.023) investig(0.020) syrian(0.016) convent(0.012) regim(0.010) ani(0.010)
15	european(0.044) kosovo(0.039) union(0.031) eu(0.030) bosnia(0.022) herzegovina(0.021) myanmar(0.015) repres(0.014) high(0.012) support(0.010)
16	day(0.069) today(0.031) year(0.030) celebr(0.030) anniversari(0.023) world(0.023) thi(0.021) mark(0.019) intern(0.016) commemor(0.014)
17	brief(0.084) live(0.074) unsc(0.065) watch(0.048) today(0.045) secur(0.027) council(0.025) meet(0.025) situat(0.021) follow(0.017)
18	itali(0.075) norway(0.049) sweden(0.039) support(0.033) fund(0.022) contribut(0.020) commit(0.019) ocean(0.018) pledg(0.013) ethiopia(0.013)
19	secur(0.040) nation(0.031) unit(0.031) intern(0.031) peac(0.027) state(0.021) council(0.020) charter(0.018) principl(0.014) world(0.014)
20	climat(0.057) chang(0.039) global(0.016) develop(0.015) action(0.014) need(0.013) sustain(0.011) impact(0.011) risk(0.011) technolog(0.010)
21	syria(0.065) syrian(0.043) humanitarian(0.023) polit(0.016) cross(0.012) regim(0.011) unit(0.011) al(0.010) peopl(0.009) continu(0.009)

22	attack(0.091) condemn(0.064) express(0.036) famili(0.032) condol(0.026) kill(0.026) strongli(0.023) victim(0.019) terrorist(0.019) civilian(0.015)
23	women(0.158) peac(0.043) particip(0.035) gender(0.026) equal(0.022) secur(0.019) agenda(0.016) girl(0.016) process(0.012) empower(0.010)
24	unit(0.061) brazil(0.047) franc(0.040) arab(0.038) estonia(0.037) emir(0.027) kingdom(0.025) albania(0.024) lithuania(0.018) arria(0.018)
25	unsc(0.151) statement(0.070) secur(0.054) debat(0.046) council(0.037) open(0.032) read(0.028) ambassador(0.023) deliv(0.021) amb(0.021)
26	palestinian(0.037) east(0.020) israel(0.019) peac(0.018) middl(0.014) isra(0.014) solut(0.013) palestin(0.013) intern(0.012) gaza(0.011)
27	elect(0.029) nation(0.022) haiti(0.020) countri(0.015) polit(0.015) support(0.014) unit(0.013) govern(0.013) ivoir(0.009) secur(0.009)
28	children(0.124) conflict(0.046) belgium(0.041) arm(0.037) protect(0.035) educ(0.033) child(0.024) school(0.023) unicef(0.014) group(0.011)
29	event(0.046) sdg(0.033) level(0.017) high(0.017) join(0.016) globalgo(0.015) host(0.014) unga(0.012) pm(0.012) tomorrow(0.011)
30	sudan(0.053) south(0.042) african(0.024) central(0.020) peac(0.019) govern(0.014) agreement(0.014) darfur(0.013) support(0.013) secur(0.011)
31	wa(0.014) state(0.014) russia(0.011) russian(0.010) countri(0.010) ha(0.010) nebenzia(0.008) osc(0.008) situat(0.007) militari(0.007)
32	committe(0.076) sanction(0.050) resolut(0.030) state(0.020) expert(0.020) implement(0.019) work(0.017) member(0.016) chair(0.016) panel(0.015)
33	switzerland(0.126) kuwait(0.078) uk(0.053) unsc(0.040) disabl(0.033) person(0.031) unit(0.024) al(0.018) kingdom(0.017) ambassadorallen(0.016)
34	terror(0.050) terrorist(0.039) counter(0.026) threat(0.019) intern(0.015) state(0.014) group(0.013) organ(0.011) nation(0.009) effort(0.009)
35	peac(0.028) somalia(0.027) mali(0.022) colombia(0.021) agreement(0.017) govern(0.015) secur(0.015) support(0.013) implement(0.012) process(0.012)
36	peopl(0.037) societi(0.035) young(0.027) civil(0.027) youth(0.024) freedom(0.023) journalist(0.016) media(0.013) social(0.012) religi(0.011)
37	afghanistan(0.091) afghan(0.042) support(0.019) peac(0.017) taliban(0.016) govern(0.014) nation(0.013) continu(0.012) unama(0.011) secur(0.011)
38	humanitarian(0.060) peopl(0.020) need(0.019) assist(0.016) million(0.015) food(0.015) access(0.012) situat(0.012) conflict(0.011) crisi(0.010)
39	libya(0.043) yemen(0.037) libyan(0.021) polit(0.020) parti(0.017) support(0.015) special(0.014) unit(0.014) yemeni(0.013) nation(0.012)
40	ukrain(0.101) russia(0.063) russian(0.027) war(0.026) ukrainian(0.025) aggress(0.013) territori(0.013) ha(0.009) continu(0.008) intern(0.008)
41	parti(0.021) polit(0.020) intern(0.018) effort(0.018) peac(0.017) dialog(0.016) countri(0.015) situat(0.013) region(0.013) commun(0.012)
42	uae(0.061) humanright(0.055) un_women(0.026) board(0.016) genderequ(0.015) nusseibeh(0.014) unfpa(0.014) amb(0.014) execut(0.014) unga(0.014)
43	right(0.177) human(0.172) refuge(0.040) traffick(0.031) violat(0.023) person(0.022) protect(0.020) migrant(0.016) commission(0.016) migrat(0.015)
44	law(0.043) intern(0.042) arm(0.032) civilian(0.032) protect(0.028) conflict(0.023) humanitarian(0.019) weapon(0.013) violat(0.010) respect(0.010)

45	peacekeep(0.064) oper(0.038) mission(0.037) unit(0.035) nation(0.033) mandat(0.017) polic(0.016) forc(0.013) train(0.013) contribut(0.012)
46	ireland(0.095) india(0.051) new(0.050) amb(0.024) perman(0.023) ambassador(0.021) york(0.021) repres(0.017) today(0.014) thi(0.013)
47	nuclear(0.050) korea(0.024) iran(0.022) weapon(0.020) prolifer(0.018) non(0.016) republ(0.016) secur(0.015) missil(0.014) peopl(0.014)
48	unsg(0.055) ban(0.051) moon(0.044) ki(0.043) congratul(0.039) elect(0.036) presid(0.018) candid(0.012) member(0.012) spain(0.011)
49	violenc(0.083) sexual(0.057) conflict(0.036) women(0.024) victim(0.016) gender(0.015) account(0.014) prevent(0.013) bas(0.013) crime(0.010)

Note: The 10 tokens listed in each row are the word stems most strongly associated with the corresponding topic. A number in parentheses denotes the strength (weight) of association.

2 Supplementary Figures

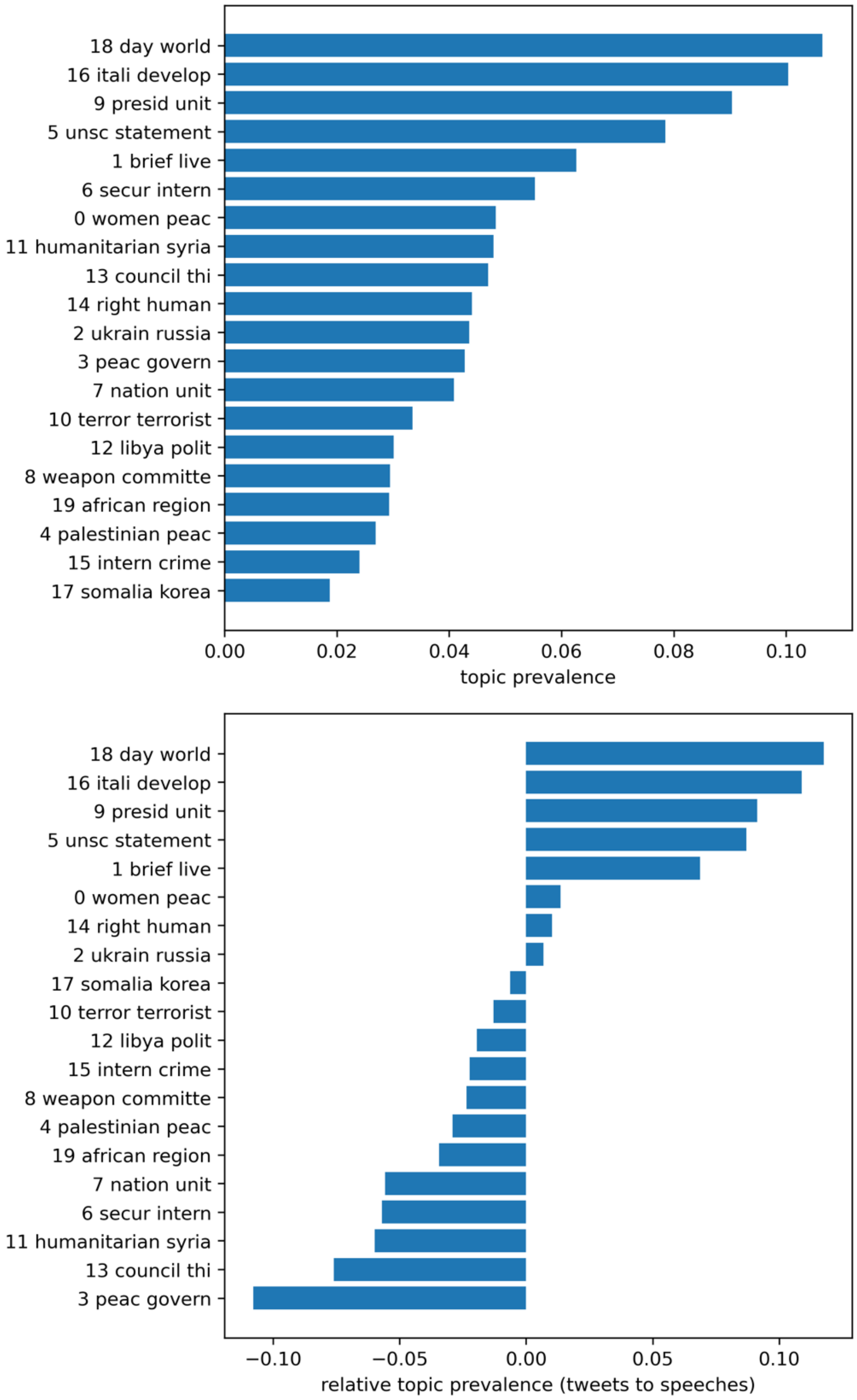

Supplementary Figure 1. Topic prevalence (*k*=20)
Top: Prevalence across the combined corpus; Bottom: Differences in prevalence between the X posts and the speeches; see Supplementary Table 3 for the content of each topic.

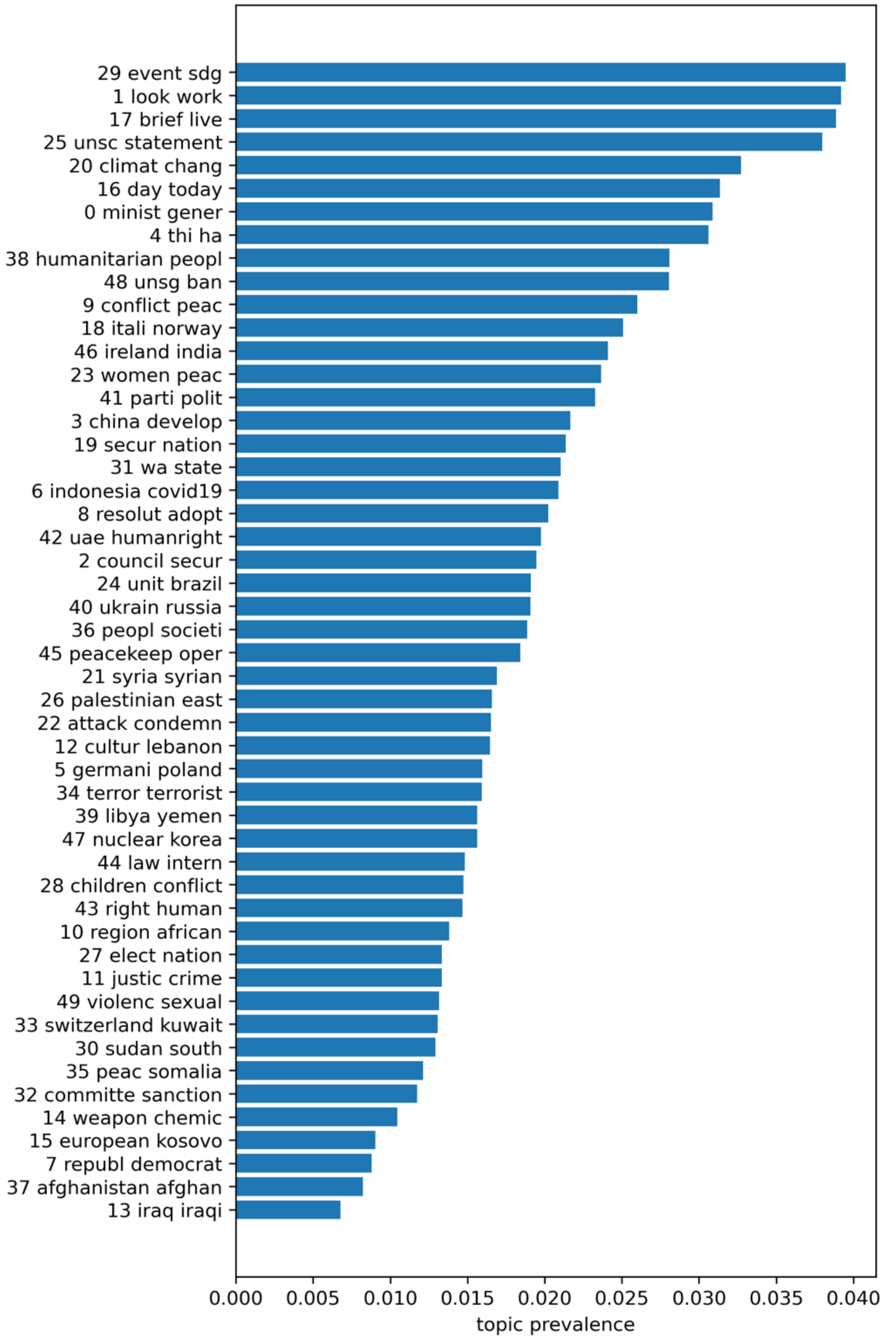
29 event sdg
1 look work
17 brief live
25 unsc statement
20 climat chang
16 day today
0 minist gener
4 thi ha
38 humanitarian peopl
48 unsg ban
9 conflict peac
18 itali norway
46 ireland india
23 women peac
41 parti polit
3 china develop
19 secur nation
31 wa state
6 indonesia covid19
8 resolut adopt
42 uae humanright
2 council secur
24 unit brazil
40 ukrain russia
36 peopl societi
45 peacekeep oper
21 syria syrian
26 palestinian east
22 attack condemn
12 cultur lebanon
5 germani poland
34 terror terrorist
39 libya yemen
47 nuclear korea
44 law intern
28 children conflict
43 right human
10 region african
27 elect nation
11 justic crime
49 violenc sexual
33 switzerland kuwait
30 sudan south
35 peac somalia
32 committe sanction
14 weapon chemic
15 european kosovo
7 republ democrat
37 afghanistan afghan
13 iraq iraqi
0.000
0.005
0.010
0.015
0.020
0.025
0.030
0.035
0.040
topic prevalence

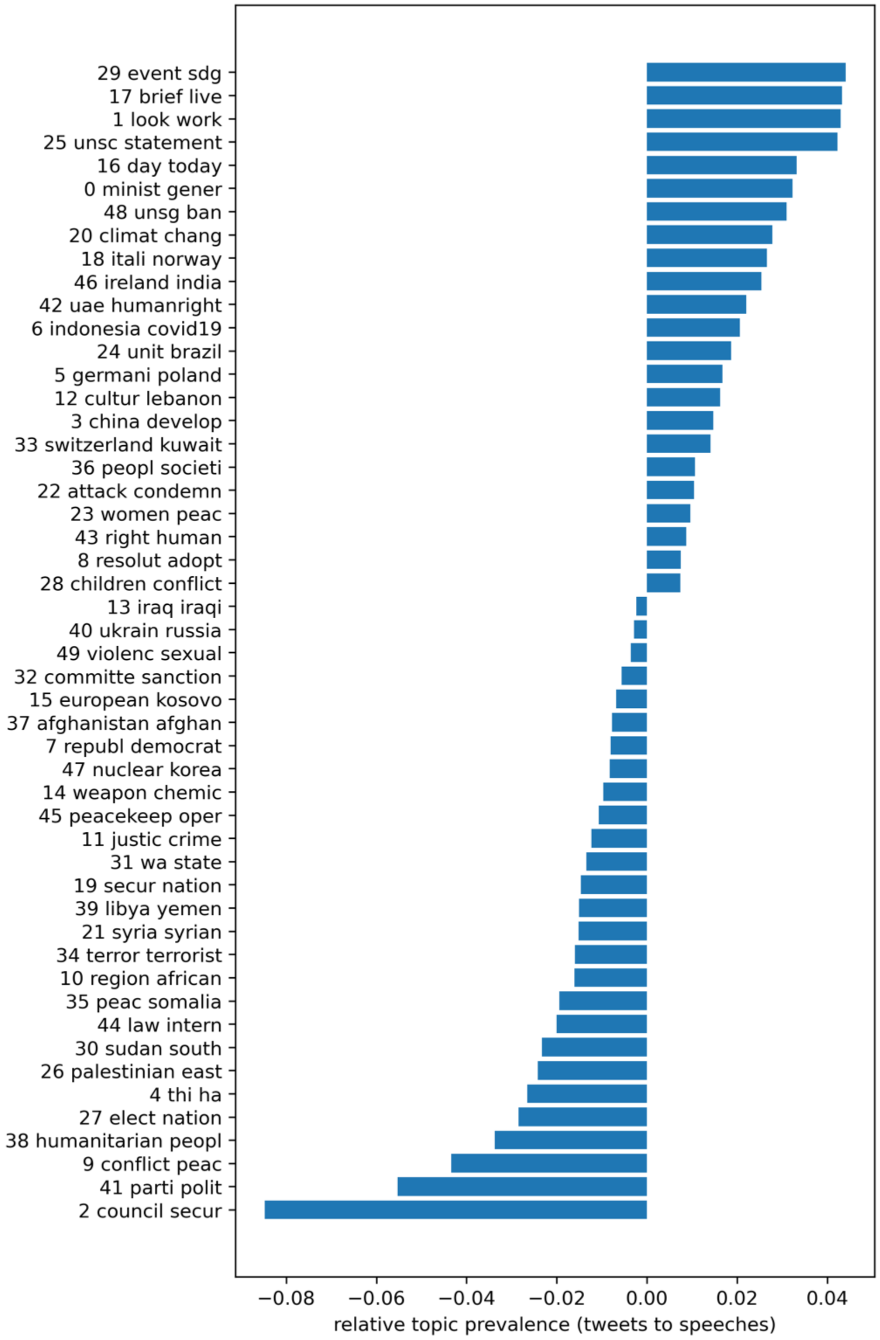

Supplementary Figure 2. Topic prevalence (*k*=50)
Top: Prevalence across the combined corpus; Bottom: Differences in prevalence between the X posts and the speeches; see Supplementary Table 4 for the content of each topic.

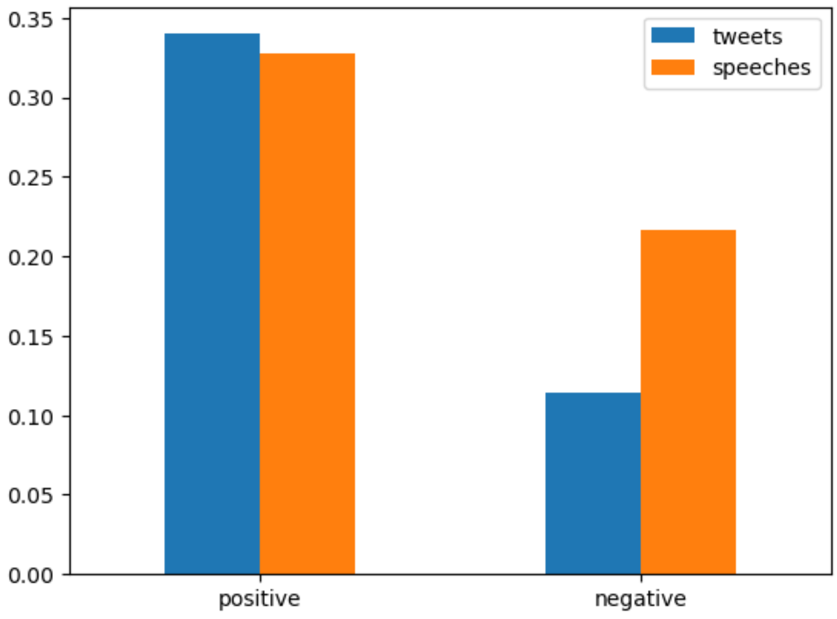

Supplementary Figure 3. Offline and online sentiment distributions (an alternative model)
See https://huggingface.co/cardiffnlp/twitter-roberta-base-sentiment-latest for the model employed.

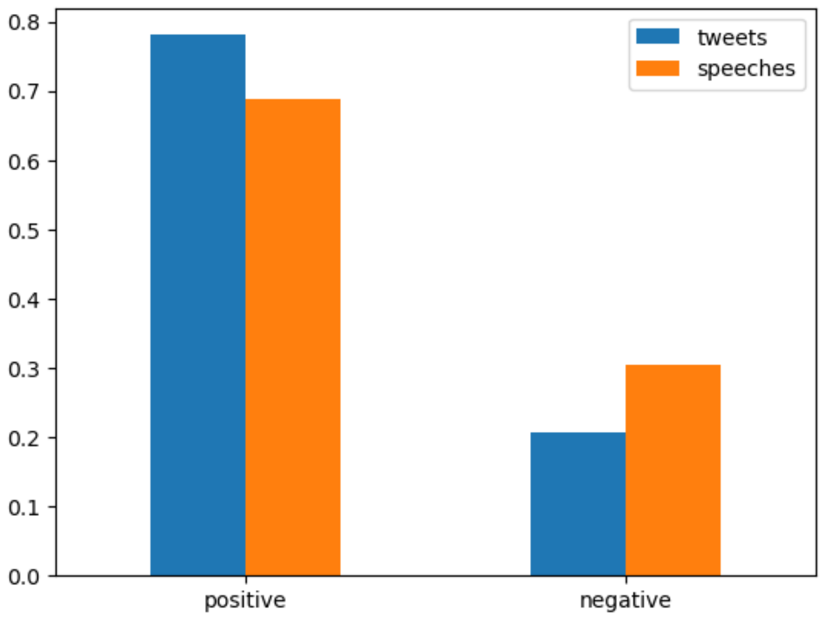

Supplementary Figure 4. Offline and online sentiment distributions in the case of China

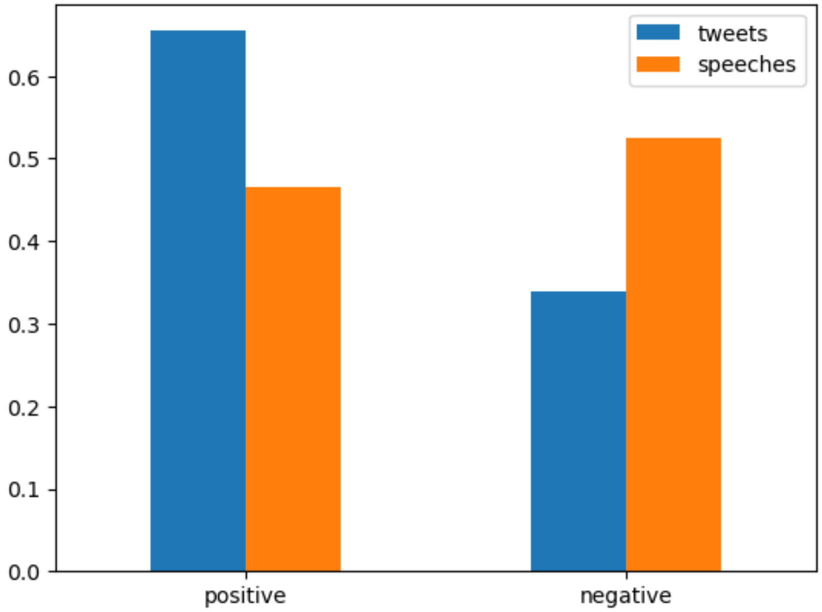

Supplementary Figure 5. Offline and online sentiment distributions in the case of the United States

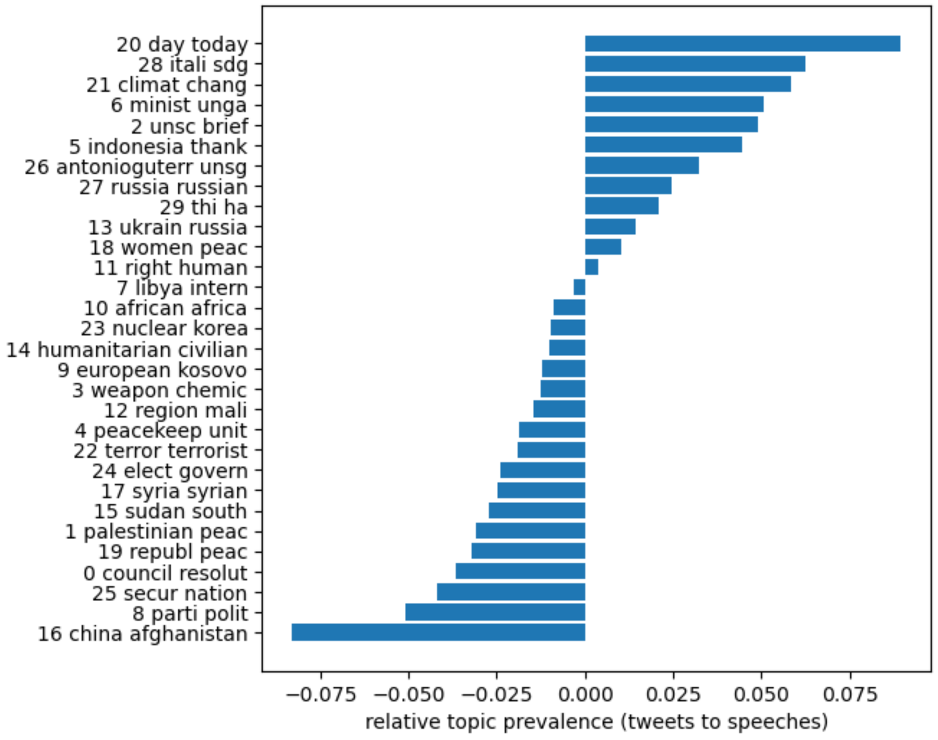
20 day today
28 itali sdg
21 climat chang
6 minist unga
2 unsc brief
5 indonesia thank
26 antonioguterr unsg
27 russia russian
29 thi ha
13 ukrain russia
18 women peac
11 right human
7 libya intern
10 african africa
23 nuclear korea
14 humanitarian civilian
9 european kosovo
3 weapon chemic
12 region mali
4 peacekeep unit
22 terror terrorist
24 elect govern
17 syria syrian
15 sudan south
1 palestinian peac
19 republ peac
0 council resolut
25 secur nation
8 parti polit
16 china afghanistan
−0.075 −0.050 −0.025 0.000 0.025 0.050 0.075
relative topic prevalence (tweets to speeches)

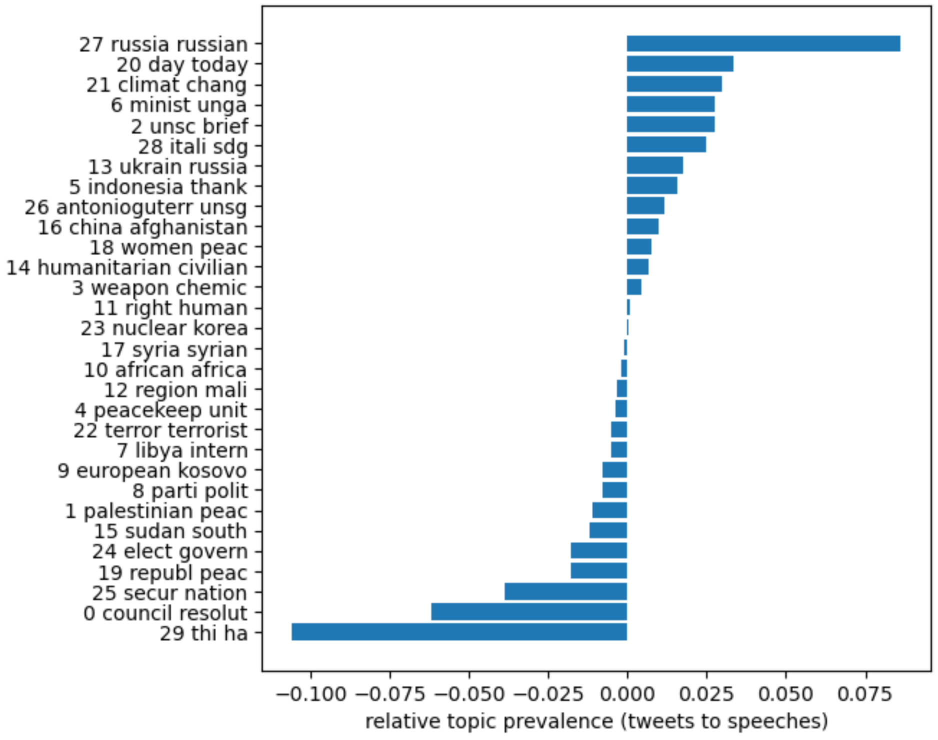
27 russia russian
20 day today
21 climat chang
6 minist unga
2 unsc brief
28 itali sdg
13 ukrain russia
5 indonesia thank
26 antonioguterr unsg
16 china afghanistan
18 women peac
14 humanitarian civilian
3 weapon chemic
11 right human
23 nuclear korea
17 syria syrian
10 african africa
12 region mali
4 peacekeep unit
22 terror terrorist
7 libya intern
9 european kosovo
8 parti polit
1 palestinian peac
15 sudan south
24 elect govern
19 republ peac
25 secur nation
0 council resolut
29 thi ha
−0.100 −0.075 −0.050 −0.025 0.000 0.025 0.050 0.075
relative topic prevalence (tweets to speeches)

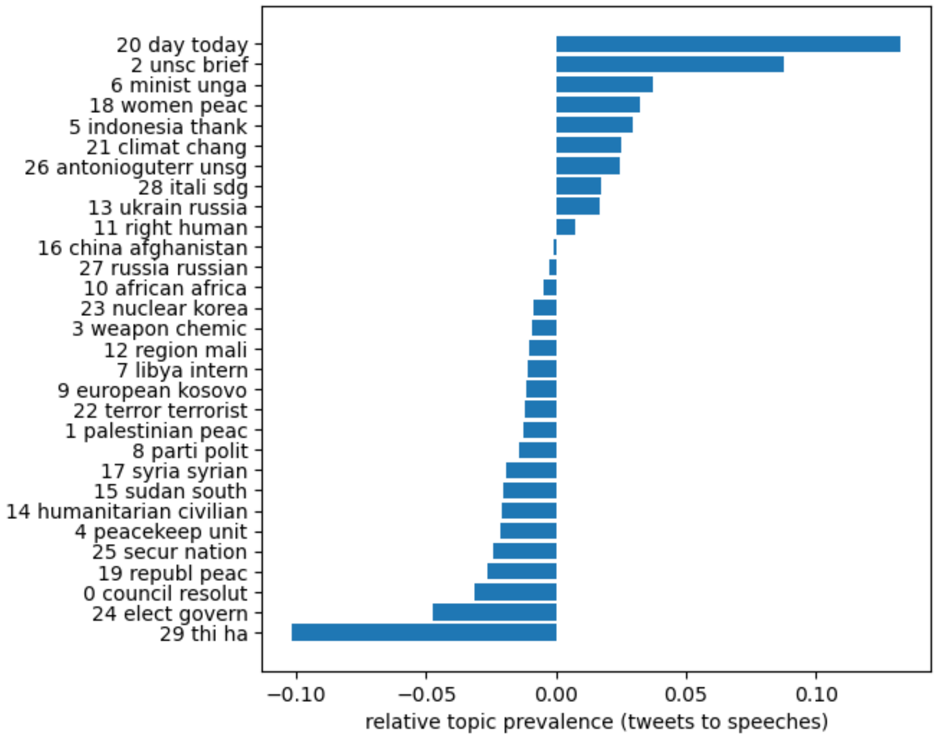

Supplementary Figure 6. Cross-national comparison of offline-online topic prevalence (k=30)
Top: China; Middle: Russia; Bottom: United States
